# Prototype multi-biomarker test for point-of-care leprosy diagnostics

**DOI:** 10.1016/j.isci.2020.102006

**Published:** 2020-12-29

**Authors:** Anouk van Hooij, Elisa M. Tjon Kon Fat, Danielle de Jong, Marufa Khatun, Santosh Soren, Abu Sufian Chowdhury, Johan Chandra Roy, Khorshed Alam, Jong-Pill Kim, Jan Hendrik Richardus, Annemieke Geluk, Paul L.A.M. Corstjens

**Affiliations:** 1 Department of Infectious Diseases Leiden University Medical Center, PO Box 9600, 2300 RC Leiden, the Netherlands; 2 Department of Cell and Chemical Biology, Leiden University Medical Center, the Netherlands; 3 Rural Health Program, The Leprosy Mission International Bangladesh, Nilphamari, Bangladesh; 4 Institute for Leprosy Research, Korean Hansen Welfare Association, Gyeonggi-do, South Korea; 5 Department of Public Health, Erasmus MC, University Medical Center Rotterdam, Rotterdam, the Netherlands

**Keywords:** Diagnostic Technique in Health Technology, Applied Microbiology, Biotechnology

## Abstract

To end the decade-long, obstinately stagnant number of new leprosy cases, there is an urgent need for field-applicable diagnostic tools that detect infection with *Mycobacterium leprae*, leprosy's etiologic agent. Since immunity against *M. leprae* is characterized by humoral and cellular markers, we developed a lateral flow test measuring multiple host proteins based on six previously identified biomarkers for various leprosy phenotypes. This multi-biomarker test (MBT) demonstrated feasibility of quantitative detection of six host serum proteins simultaneously, jointly allowing discrimination of patients with multibacillary and paucibacillary leprosy from control individuals in high and low leprosy endemic areas. Pilot testing of fingerstick blood showed similar MBT performance in point-of-care (POC) settings as observed for plasma and serum. Thus, this newly developed prototype MBT measures six biomarkers covering immunity against *M. leprae* across the leprosy spectrum. The MBT thereby provides the basis for immunodiagnostic POC tests for leprosy with potential for other (infectious) diseases as well.

## Highlights

• Prototype MBT that quantitatively detects six host-derived biomarkers is developed
• The immunopathological spectrum of leprosy is ideally suited to evaluate the MBT
• MBT discriminated patients with leprosy from controls in a high and non-endemic area
• Application of the MBT using low invasive fingerstick blood is technically feasible

## Graphical Abstract

**Figure:**
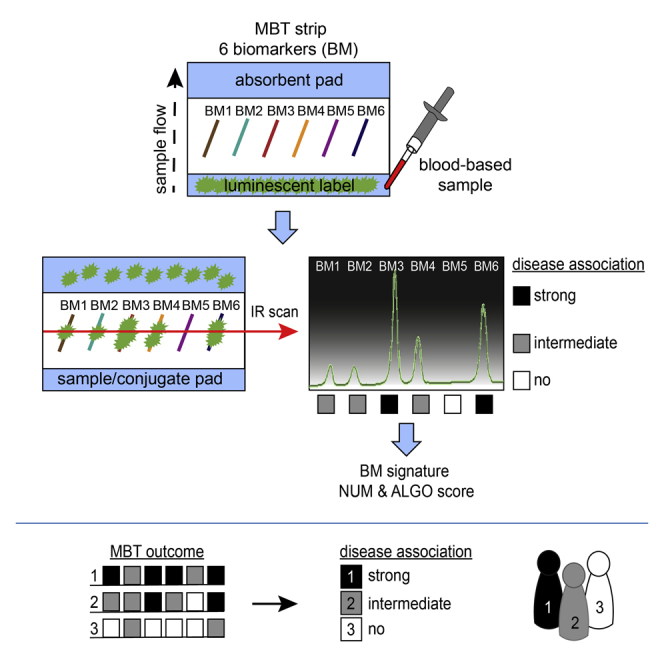

## Introduction

For over a decade, the annual number of newly detected leprosy cases has stagnated around 200,000 including children (World Health Organisation, 2019). This indicates that transmission of the causative agent of leprosy, *Mycobacterium leprae (M. leprae)*, is still ongoing. Leprosy can be effectively cured by multidrug therapy (MDT), and early identification and treatment of patients with leprosy prevents irreversible nerve damage correlated with advanced stages of the disease (Thangaraju and Venkatesan, 2020). Prevention of disability reduces health as well as socioeconomic burden on leprosy-affected individuals, as their visible handicaps can lead to loss of income or unemployment due to social stigma and exclusion. Currently, leprosy diagnosis is based on clinical symptoms requiring well-trained clinicians. As a result of the declaration by the World Health Organization (WHO) in 2000 that the global target of leprosy elimination had been reached (World Health Organisation, 2002), leprosy control activities received considerably less attention and leprosy care was integrated in general healthcare programs. This leads to diminished leprosy expertise among clinicians which currently results in frequent missed or delayed diagnosis (Smith et al., 2015). Undiagnosed patients and *M. leprae*-infected individuals (yet) without clinical symptoms are likely to contribute significantly to the ongoing transmission (Pedrosa et al., 2018), which is emphasized by the fact that 75% of the new leprosy cases in high endemic areas cannot be directly attributed to known index cases (Richardus et al., 2005; Ortuno-Gutierrez et al., 2019a). Implementation of diagnostic tests specific for *M. leprae* infection in contact and population surveys will allow the identification of *M. leprae*-infected individuals as target for post-exposure prophylaxis (PEP), as well as detection of early stage leprosy for timely treatment (Blok et al., 2018; GPZL, 2019). Such diagnostic tests are not yet available (GPZL, 2019; Smith et al., 2016). Moreover, in order to implement novel tools in leprosy endemic areas, which are often resource-limited settings, diagnostic tests need to be available in a user- and field-friendly, rapid test format.

Leprosy has a wide spectrum of clinical manifestations which are closely related to the host immune response against *M. leprae*. In patients with multibacillary (MB) leprosy (individuals with high bacillary loads), IgM antibody responses to phenolic glycolipid-I (PGL-I), a cell wall component of *M. leprae*, are frequently detected (van Hooij et al., 2017). In paucibacillary (PB) leprosy, this antibody response is generally absent, but instead, biomarkers of (Th1-)cell-mediated immunity are observed (van Hooij et al., 2018, 2019). Examining the anti-*M. leprae* antibody response only is therefore not sufficient to identify patients at both sides of the leprosy spectrum but requires detection of multiple biomarkers specific for humoral as well as cellular immunity (Geluk, 2013a). Recently, we identified host biomarkers associated with leprosy in *M. leprae* antigen-stimulated whole blood assays (WBAs) and plasma from a leprosy endemic population in Bangladesh. A host biomarker signature of αPGL-I IgM, IP-10, CRP, ApoA1, and S100A12 was identified, covering both the humoral and cellular pole of the immunopathologic leprosy spectrum (van Hooij et al., 2019; Nausch and Jacobsen, 2019). High αPGL-I IgM, IP-10, and CRP levels, relative to controls, were associated with MB leprosy, whereas ApoA1 and S100A12 levels were critical for identification of both patients groups. For patients with PB leprosy, ApoA1 was identified as the most important biomarker (van Hooij et al., 2019). ApoA1 and S100A12 levels also differentiated highly exposed contacts from endemic controls (ECs), identifying potentially *M. leprae*-infected individuals (van Hooij et al., 2020). In addition, CCL4 showed added diagnostic value in overnight stimulated WBA samples, particularly for patients with PB leprosy (van Hooij et al., 2019) and was also associated with *M. leprae* infection among household contacts (van Hooij et al., 2020).

Utilizing the unique up-converting reporter particles (UCPs), individual lateral flow (LF) test strips for separate detection of each of the five identified biomarkers were previously developed and applied to several cohorts from different geographic regions (van Hooij et al., 2016, 2018, 2019). UCP-LF is virtually background free as the up-conversion upon excitation with infrared light does not occur in nature. This prevents autofluorescence with other assay components, providing a rapid and highly sensitive point-of-care (POC) test format (Corstjens et al., 2005; Sedlmeier et al., 2016; Tanke et al., 2014). In contrast to most POC tests (Buhrer-Sekula et al., 2003), the results generated by UCP-LF tests are quantitative. This allows cross-sectional comparison of test groups, as well as intra-individual longitudinal monitoring, at the POC level.

Aiming at user- and field-friendly test applications, we developed a multi-biomarker test (MBT) strip that allows simultaneous detection of these six biomarkers on one strip rather than separate strips for each biomarker. To demonstrate feasibility of this MBT to identify patients with leprosy and *M. leprae*-infected individuals, we analyzed banked plasma and serum samples of patients with leprosy from a highly endemic area in Bangladesh (Richardus et al., 2013), as well as an area in South Korea, that has reached the WHO elimination target (registered prevalence of less than 1 case per 10,000 population) in 1984 (Lee et al., 2015) but still reports new (import) leprosy cases annually (World Health Organisation, 2019). Finally, we pilot tested the MBT in Bangladesh collecting and directly testing fingerstick blood (FSB) samples from patients with leprosy and their contacts in the field to assess POC application of the MBT.

## Results

### The MBT format

**Figure 1:**
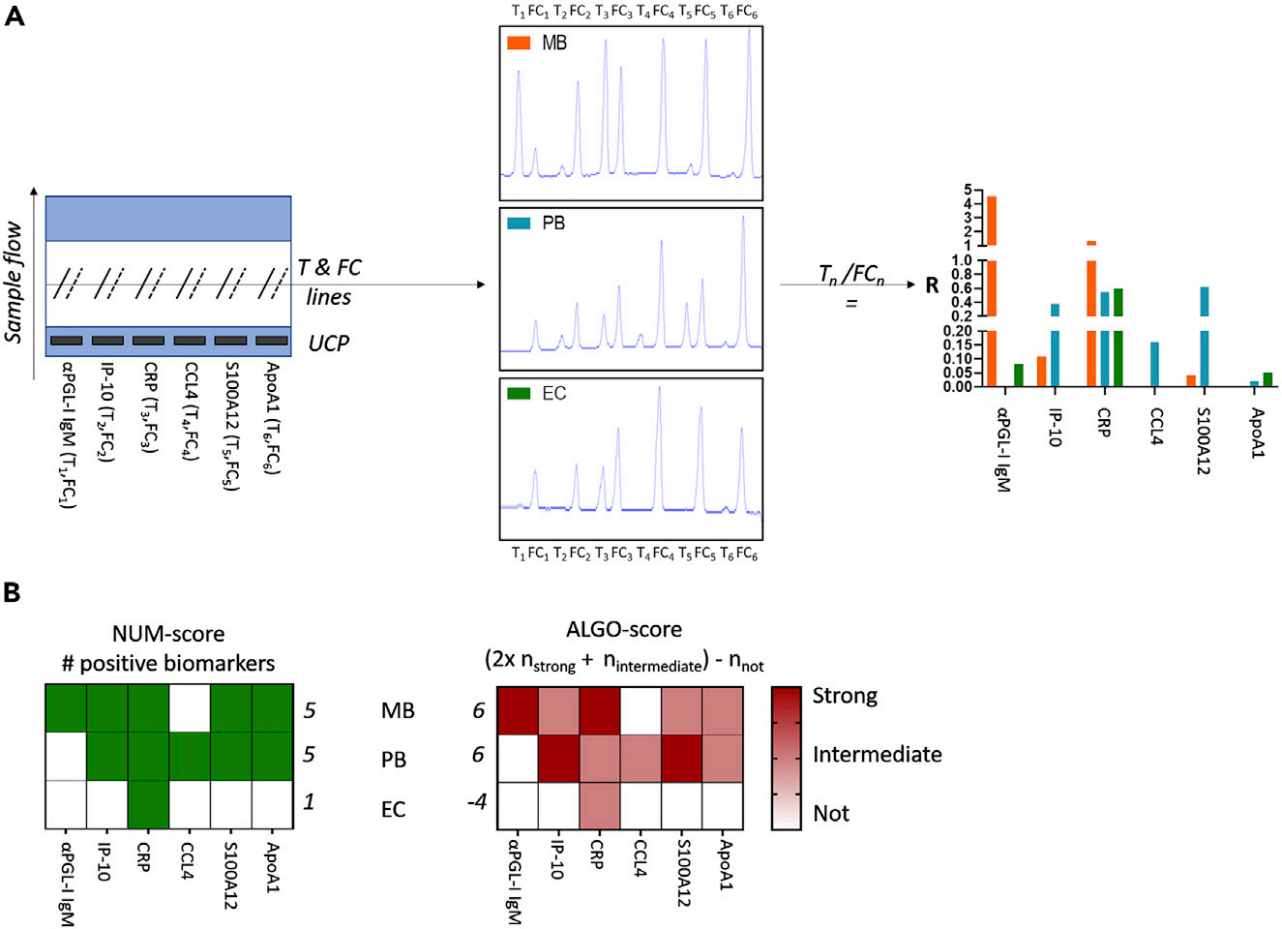
MBT schematic overview and scoring (A) The MBT strip consists of 12 parallel lines, of which six are test lines (T_n_) and six are flow controls (FC_n_). Each T_n_ and FC_n_ pair measures a single biomarker: αPGL-I IgM, IP-10, CRP, CCL4, S100A12, ApoA1. The up-converting reporter particles (UCP) are incorporated in the sample pad. The strip is read using a portable reader perpendicular to the sample flow. The test readout is a pattern of peaks, showing the signal of each of the 12 lines resulting in a ratio value (R) per biomarker (T_n_/FC_n_). R values for each biomarker are displayed for plasma samples of a patient with multibacillary leprosy (MB; orange) and an endemic control (EC; green) and an *M. leprae* antigen-stimulated whole blood assay sample of a patient with paucibacillary leprosy (PB; blue). (B) Two scores were calculated using the R values, the NUM score and the ALGO score. The NUM score is the sum of positive biomarkers per individual (green; R value above the cutoff). The ALGO score is based on an algorithm that contributes higher weights to R values associated with disease and was calculated using the median R values of the individual biomarkers for the patients with leprosy.

MBT strips comprising six test lines (biomarkers) with their respective flow control lines were produced using the sequence of biomarkers as indicated in Figure 1A. For this study, a predefined five-biomarker signature for leprosy (αPGL-I IgM, IP-10, CRP, S100A12, and ApoA1 (van Hooij et al., 2019)) was incorporated in the MBT strip format. As we envisage use of MBT strips both as POC tests and as user-friendly rapid tests for overnight stimulated whole blood samples, CCL4 as a biomarker for PB leprosy (van Hooij et al., 2016, 2018, 2019) was included as the sixth biomarker. The readout provided by the luminescent reporter technology (UCP) is indicated as the R value, for each biomarker individually. The R value is a relative value that quantifies the difference between the signal intensity of the test line and flow control. For each biomarker, this relative value can be converted to concentrations by generating a standard curve using known (recombinant) biomarker concentrations.

Application of the MBT to representative samples of EC and clinically diagnosed patients with MB and PB leprosy clearly showed the difference in peak height and concomitant R values between the plasma samples of the patient with MB leprosy and the EC, as well as the WBA sample of the patient with PB leprosy (Figure 1A). Importantly, overnight stimulation with *M. leprae* antigens, as demonstrated for the patient with PB leprosy, increased CCL4 levels which were undetectable in unstimulated samples/sera (van Hooij et al., 2019). Moreover, the dichotomy in αPGL-I IgM between MB and PB unambiguously confirmed the presence of antibodies in the former leprosy type and absence in the latter.

The MBT format thus enabled simultaneous, quantitative detection of six biomarkers in one test (Figure 1A), thereby representing a unique feature for user-friendly lateral flow assays. To explore scoring procedures for the readout, we defined and evaluated the NUM score and ALGO score. The NUM score is based on the sum of the number of positive biomarkers detected in the MBT providing a quick and easy-to-interpret readout. This required determination of a cutoff R value to discriminate patients with leprosy from controls, which was done using the Youden's index (Fluss et al., 2005) for each individual biomarker. The ALGO score was based on an algorithm that tentatively indicates an association of the R values with disease (Figure 1B). Using the biomarker median R value of the patient group, individual R values were classified as strongly, intermediately, or not associated with disease. Figure 1B illustrates both scoring methods, showing an MB and PB sample with higher NUM and ALGO scores as compared to the EC.

### MBT evaluation in two cohorts with varying leprosy endemicity

To further explore the MBT performance, MBT strips were applied to banked plasma samples from Bangladesh and banked sera from South Korea.

#### Bangladesh (high endemic area)

Since the biomarkers studied here are generally not present in the same concentration range in blood, the optimal sample dilution per biomarker (10-fold and 1000-fold) was first determined. Results indicated that the biomarkers present in high concentrations (ApoA1, CRP, and αPGL-I IgM) based on previously obtained ELISA data (van Hooij et al., 2019) distinguished patients with leprosy from controls effectively using 1000-fold dilutions, whereas for detection of IP-10 and S100A12, 10-fold dilutions were required (Figure S1). CCL4 could not be detected in unstimulated plasma samples, in line with what we have observed previously (van Hooij et al., 2019). For the other five individual biomarkers, areas under the curve (AUCs) observed in this cohort were comparable to results from an earlier study (van Hooij et al., 2019) using the same plasma samples but with multiple singleplex UCP-LF strips (Figure S2).

**Figure 2:**
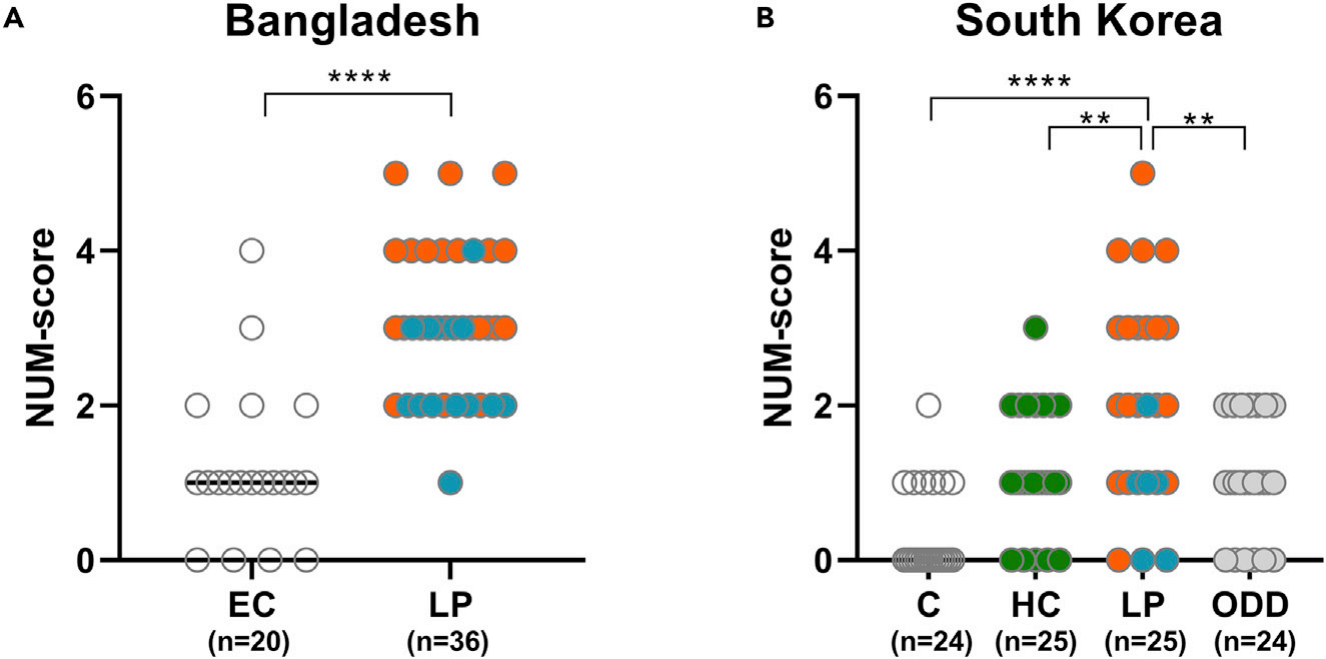
The MBT NUM score identifies patients with leprosy The levels of αPGL-I IgM, IP-10, CCL4, CRP, S100A12, and ApoA1 were assessed by the MBT strip. The NUM score, indicating the number of positive biomarkers based on the ratio value, was calculated per individual (*y* axis). (A) NUM scores observed in the cohort from Bangladesh (plasma) comparing patients with leprosy to healthy endemic controls (ECs). (B) NUM scores in the South Korean cohort comparing patients with leprosy to healthy controls (C), household contacts (HCs), or patients with other dermatological diseases (ODDs). Group differences were determined using the Mann-Whitney U test; the statistical significance level used was p ≤ 0.05. ∗p ≤ 0.05, ∗∗p ≤ 0.01, ∗∗∗p ≤ 0.001, ∗∗∗∗p ≤ 0.0001. Patients with multibacillary leprosy are indicated with orange dots, patients with paucibacillary leprosy with blue dots, HCs with green dots, patients with ODDs with gray dots, and healthy controls with white dots.

The NUM score as determined previously with singleplex UCP-LF strips accurately distinguished patients with leprosy from EC (AUC:0.93) (van Hooij et al., 2019). Application of the NUM score to the MBT results showed a similar AUC (AUC: 0.9: p < 0.0001; Figure 2A). This score thus performed equally well for the MBT as for singleplex strips, signifying the potential of this MBT readout to identify patients with leprosy in endemic areas.

#### South Korea (non-endemic)

The MBT was also evaluated using serum samples from a South Korean cohort (Figure S3). Application of the NUM score to the MBT data significantly discriminated patients with leprosy from healthy controls living in that area (AUC: 0.88; p < 0.0001) (Figure 2A). Furthermore, patients with leprosy showed significantly higher NUM scores than contacts of patients with leprosy or patients with other dermatological diseases (ODDs) (household contact [HC]: p = 0.0079; ODD: p = 0.003; Figure 2B). These data indicate the applicability of the MBT to identify patients with leprosy also in a non-endemic area.

**Figure 3:**
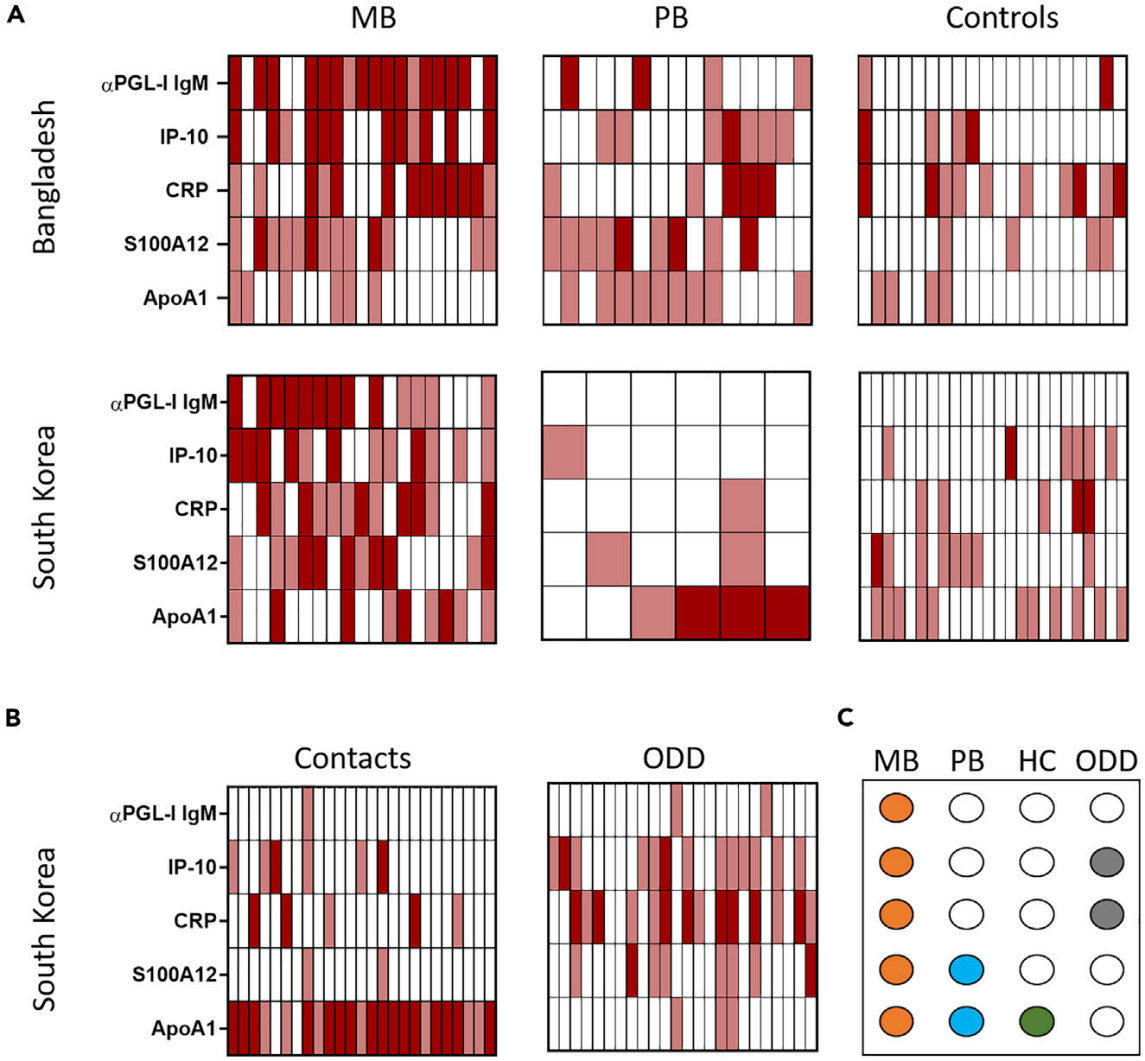
Stratification of biomarker levels using the MBT readout Heatmap indicating per individual the αPGL-I IgM, IP-10, CRP, S100A12, and ApoA1 R values classified in three groups based on the association with disease as strong (dark red), intermediate (pink), or not associated (white) in the Bangladeshi and South Korean cohort. Strong association: R ≥ 2x median of the patient group, intermediate association: median of the patient group ≤ R < 2x median of the patient group, no association: R < median of the patient group (Table S1). (A) R value classification of biomarkers in patients with multibacillary (MB) leprosy (Bangladesh: 21; South Korea: 19), patients with paucibacillary (PB) leprosy (Bangladesh: 15; South Korea: 6), and healthy controls (Bangladesh: 20; South Korea: 24). (B) R value classification of biomarkers in household contacts (HCs; n = 25) and patients with other dermatological diseases (ODDs; n = 25) (South Korean cohort). (C) Dots indicating which biomarker showed a different pattern relative to controls in patients with MB (orange) and PB (blue) leprosy, HC (green), and patients with ODDs (gray).

Although the NUM score performed equally well in both cohorts to differentiate patients with leprosy from healthy controls, it transformed the quantified MBT readout to a qualitative result (positive or negative) per biomarker. Apart from αPGL-I IgM, the other five MBT biomarkers are also present in unexposed, healthy individuals but the R values observed differed between patients and controls. Thus, to evaluate the difference in biomarkers between test groups, we stratified R values as strongly, intermediately, or not associated with disease, based on the median R value per biomarker determined for the group of patients with leprosy in each country (Table S1). This showed that the earlier observed pattern for patients with MB leprosy of high αPGL-I IgM, CRP, and IP-10 R values (van Hooij et al., 2019) was confirmed in both the Bangladeshi and South Korean cohorts. Similarly, MBT data of both cohorts showed that the ApoA1 R values in patients with PB leprosy differed from those in healthy controls (Figure 3). Interestingly, contacts of patients with leprosy in South Korea showed ApoA1 R values similar to those of patients with PB leprosy. This indicates not only the potential of this biomarker for discriminating PB from ODDs but also to detect *M. leprae* exposure/infection. An overview of the biomarkers differentiating controls from patients with MB and PB leprosy, HCs, and patients with ODDs indicates that assessing a combination of biomarkers is essential to allow proper interpretation of the MBT outcome (Figure 3C).

**Figure 4:**
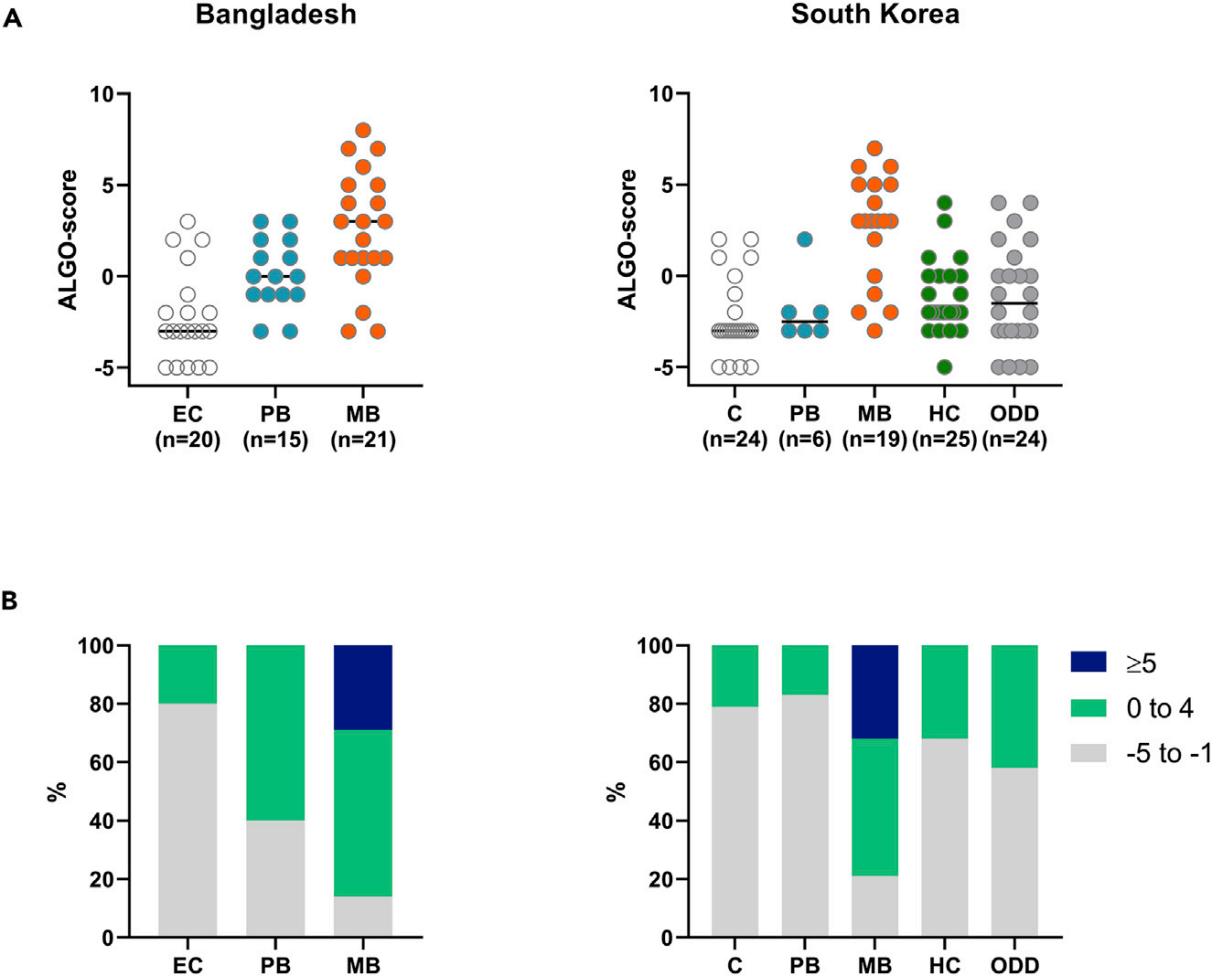
The MBT ALGO score reflects disease severity and bacterial load The ALGO score is based on an algorithm that contributes higher weights to R values associated with disease (Figure 1B). Based on the median R value of the patient group, for each biomarker, R values were classified as strongly, intermediately, or not associated with disease. The ALGO score was set as 2× the number of strong biomarkers (2× n_strong_), plus the number of intermediate biomarkers (n_intermediate_), minus the number of biomarkers not associated with disease (n_not_) ((2∗nbiomarker^strong^ + nbiomarker^intermediate^)-nbiomarker^not^). (A) ALGO scores observed in the Bangladeshi and South Korean cohorts per test group. Patients with untreated multibacillary (MB) leprosy (orange), patients with untreated paucibacillary (PB) leprosy (blue), household contacts (HCs; green), patients with other dermatological diseases (ODDs; gray), and healthy controls ((E) C; white) are represented. (B) The percentage of individuals per test group with ALGO scores ranging from −5 to −1 (gray), from 0 to 4 (green), or ALGO scores ≥ 5 (blue) for the Bangladeshi plasma (left) and South Korean serum (right) cohort.

To reflect the effect of the observed patterns in individual biomarker R values in the MBT results, we assessed the second scoring method. This ALGO score showed a clear gradient from patients with MB leprosy to patients patients with PB leprosy and healthy controls in Bangladesh (Figure 4). For the South Korean cohort, however, the ALGO score of patients with PB leprosy did not differ from the scores observed in controls and contacts. Interestingly, patients with MB leprosy clearly showed the highest ALGO scores, and scores ≥ 5 were uniquely observed in this patient group (Figure 4B). This observation implicates that the ALGO score is associated with bacterial load in patients with leprosy.

In summary, the MBT accurately detected multiple biomarkers using a single test strip and allowed detailed assessment of biomarkers in blood samples. Two scoring methods were explored to interpret the MBT results, the easy-to-use NUM score to indicate the number of positive biomarkers and the more quantitative ALGO score reflecting the number of biomarkers per individual displaying R values strongly, intermediately, or not associated with disease. Application of these scoring methods facilitates interpretation of the quantitative MBT readout to identify patients with leprosy and *M. leprae*-infected individuals.

### MBT evaluation in fingerstick blood

A pilot test to evaluate the use of FSB combined with the MBT was performed in Bangladesh aiming at future POC application. All 42 FSB samples were collected from patients and contacts visiting the field hospital on the same day. Analysis of the MBT strips by a portable UCP reader showed that all biomarkers could be clearly detected in FSB enabling the determination of R values. R values similar to those in plasma/sera samples were obtained (Figure S4), demonstrating that hemoglobin formed by hemolysis did not hamper the UCP signal. Importantly, like in sera and plasma samples, higher NUM scores were more frequently observed in FSB of patients with leprosy compared to HCs (Figure S4). Hence, application of the MBT using low invasive FSB samples is technically feasible at low-resource settings providing potential for MBT use at POC.

## Discussion

This study provides proof of concept for the use of the MBT platform, thereby representing, to the best of our knowledge, the first demonstration of a diagnostic tool simultaneously and quantitatively detecting multiple host biomarkers with a user-friendly test easily applicable with FSB in the field.

Leprosy is ideally suited as a model disease to test this platform due to the close parallel between the ability of the host to establish effective immunity to *M. leprae* and the inter-individual variability in clinical manifestations, ranging from self-limited (PB) disease with a predominant Th1 response to disseminated (MB) disease characterized by extensive anti-*M. leprae* antibody titers (Geluk, 2013a, 2013b, 2018). This study showed POC testing of a biomarker signature covering humoral and cellular immune responses against *M. leprae* (van Hooij et al., 2019). The combination of six biomarkers in this new strip format in a single MBT device avoids running six individual tests and as such is a major step forward toward POC near-patient applications. Moreover, the procedure is less prone to error as the automated reader will immediately provide the MBT result; this would be much more complicated when running the six individual tests in sequence. The six-marker MBT strip provided similar test results as previously obtained with individual UCP-LF strips for each of the biomarkers separately (van Hooij et al., 2019). This clearly demonstrated technical feasibility of this new diagnostic platform.

Besides, enabling detailed evaluation of six biomarkers individually, the MBT allows combined analysis of multiple biomarkers as part of a biomarker signature. To allow for scoring, two methods were explored which are independent of each other. The NUM score, indicating the number of biomarkers with a value above the biomarker-specific cutoff, allowed discrimination of patients with leprosy from their contacts and healthy individuals. The ALGO score represents a more direct quantitative score linking the relative biomarker R values with leprosy disease. Irrespective of leprosy endemicity, patients with MB leprosy showed the highest ALGO scores, confirming the association of these MBT-implemented biomarkers with disease severity and bacterial load (van Hooij et al., 2019). Both scores are an example of the ample possibilities to analyze the MBT readout. As described previously, standard curves can also be generated to convert the quantifiable R values to absolute concentrations (Corstjens et al., 2011, 2016).

Selection of a suitable scoring method depends on the aim of the study. The NUM score provides a quick interpretation of the test result suitable for large-scale screening studies, for instance, to identify *M. leprae*-infected individuals that contribute to the perpetuating transmission. HCs of patients with MB leprosy are at the highest risk of acquiring *M. leprae* infection (Bakker et al., 2006; Goulart et al., 2008; Sales et al., 2011) and thus represent candidates for preventive drug administration in multiple studies (Barth-Jaeggi et al., 2016; Mieras et al., 2018; Ortuno-Gutierrez et al., 2019b; Richardus et al., 2020; Tiwari et al., 2020) to prevent progress to leprosy disease, as well as decrease transmission. Since June 2018, the WHO guidelines for leprosy control have included single-dose rifampicin as PEP for leprosy prevention (World Health Organization, 2018). The MBT could aid in the identification of *M. leprae*-infected individuals eligible for PEP to allow a more efficient and better targeted drug administration approach.

For personalized diagnostics and monitoring of the treatment response, the more detailed evaluation by the ALGO score could be informative. On the other hand, the MBT can be useful as adjunct diagnostic for patients presenting with symptoms suggestive of leprosy in both leprosy endemic and non-endemic countries. Identification of patients with PB leprosy lacking anti-*M. leprae* antibodies is challenging using the currently available diagnostic methods leading to delayed or misdiagnosis. In Bangladesh, the MBT result separated patients with PB leprosy clearly from ECs, although in the low endemicity setting in South Korea, the current biomarker signature could not distinguish the small-sized cohort of patients with PB leprosy (n = 6). Separate evaluation of biomarkers, however, indicated that ApoA1 differed significantly in these patients with PB leprosy from controls, corroborating the potential of ApoA1 as a biomarker for PB leprosy (van Hooij et al., 2019). *M. leprae*-exposed HCs in South Korea showed a similar ApoA1 response as the patients with PB leprosy, as observed previously in Bangladesh (van Hooij et al., 2020). In contrast to our findings in Bangladesh (van Hooij et al., 2020), R values of S100A12 in contacts and patients with PB leprosy were similar to those of healthy controls in South Korea. Leprosy is no longer endemic in this country; it has to be taken into account that the frequency of exposure to *M. leprae*, as well as other environmental pathogens, can definitely influence biomarker levels, stressing the importance of quantitative measurements.

For global application including identification of patients with PB leprosy, the currently implemented biomarker signature will need fine-tuning and evaluation in large cohorts is warranted. The flexible MBT format allows replacement of biomarkers upon identification of additional candidate markers. To identify new biomarkers, especially for PB leprosy, the scope can be widened from the broadly studied immune markers to metabolic markers, which contribute to leprosy pathogenesis as well (Mayboroda et al., 2016; Cruz et al., 2008; Silva et al., 2017). New techniques (Koeken et al., 2020; Mourits et al., 2020) to identify disease markers in HCs developing PB leprosy in high throughput fashion are currently explored by us.

An important advantage of the MBT is its field applicability, ensuring implementation in low-resource settings. Furthermore, its flexible format also enables the application of the MBT to other diseases for which diagnosis will benefit from the quantitative detection of multiple biomarkers simultaneously. Serum biomarker signatures have been described, for example, for tuberculosis (Chegou et al., 2016; Jacobs et al., 2016), rheumatoid arthritis (Li et al., 2018), and inflammatory bowel disease (Knutson et al., 2013). More recently, it was also described for patients with COVID-19 that cytokines play an important role in determining the outcome of infection besides SARS CoV-2-specific antibodies (Grifoni et al., 2020; Sekine et al., 2020).

In this study, we demonstrated the technical feasibility and applications of the MBT platform for leprosy diagnostics by successfully implementing host biomarkers covering a well-defined biomarker signature for leprosy, on one MBT strip. Moreover, the MBT was not only compatible with plasma and serum but allowed POC testing with FSB samples. Thus, the MBT format represents a step forward in the development of the urgently needed immunodiagnostic POC test for detection of *M. leprae* infection and early stage leprosy.

### Limitations of the study

We acknowledge several limitations to the findings in this study: of the patients with leprosy studied for the fingerstick blood assay we some were already treated with MDT. This cohort was therefore less homogeneous and showed variable associations with disease depending on the duration of treatment. As this study aimed at developing a test platform rather than evaluating a biomarker signature, relatively small sample sizes were tested. For the same reason, direct comparison of FSB and plasma/serum was not included, and intra-individual differences in biomarker R values in these different samples could therefore not be determined.

### Resource availability

#### Lead contact

Further information and requests for resources and reagents should be directed to A.geluk@lumc.nl.

#### Material availability

There are restrictions to the availability of MBT due to the fact that the MBT currently is an in house produced test.

#### Data and code availability

The published article includes all data generated or analyzed during this study.

## Methods

All methods can be found in the accompanying Transparent Methods supplemental file.

## Supplemental information

Document S1. Transparent methods, Figures S1–S4, and Table S1
