# Supplementary material for: Prototype multi-biomarker test for point-of-care leprosy diagnostics
Source: iScience. 2020 Dec 29;24(1):102006. doi: 10.1016/j.isci.2020.102006 (PMC7807156; doi:10.1016/j.isci.2020.102006)
Supplement: Document S1. Transparent methods, Figures S1–S4, and Table S1 [file mmc1.pdf]

iScience, Volume *24*

Supplemental Information

Prototype multi-biomarker test for point-of-care leprosy diagnostics

Anouk van Hooij, Elisa M. Tjon Kon Fat, Danielle de Jong, Marufa Khatun, Santosh Soren, Abu Sufian Chowdhury, Johan Chandra Roy, Khorshed Alam, Jong-Pill Kim, Jan Hendrik Richardus, Annemieke Geluk, and Paul L.A.M. Corstjens

Supplemental information

Prototype multi-biomarker test strip for point-of-care leprosy diagnostics

Anouk van Hooij[1], Elisa M. Tjon Kon Fat[2], Danielle de Jong[2], Marufa Khatun[3], Santosh Soren[3], Abu Sufian Chowdhury[3], Johan Chandra Roy[3], Khorshed Alam[3], Jong-Pill Kim[4], Jan Hendrik Richardus[5], Annemieke Geluk[1,6*] and Paul L.A.M. Corstjens[2*]

[1]*Department of Infectious Diseases Leiden University Medical Center, The Netherlands;*

[2]*Department of Cell and Chemical Biology, Leiden University Medical Center, The Netherlands*.

[3]*Rural Health Program, The Leprosy Mission International Bangladesh, Nilphamari, Bangladesh.*

[4]*Institute for Leprosy Research, Korean Hansen Welfare Association, Gyeonggi-do, South Korea.*

[5]*Department of Public Health, Erasmus MC, University Medical Center Rotterdam, Rotterdam, The Netherlands.*

[6]*Lead Contact*

[*]These authors contributed equally to this work

Supplementary Tables

Supplementary Table S1: Study cohorts related to Figures 2 -4.

	Group	N	BI (Mean)	Age range (years)	Male/Female (%)	MDT
Bangladesh (plasma)	*MB*	21	2.9+	18 to 60	91/9	0 (0%)
	PB	15	0	19 to 56	71/29	0 (0%)
	EC	20	NA	20 to 41	40/60	NA
	**Biomarker characteristics**	**αPGL-I IgM**	**IP-10**	**CRP**	**S100A12**	**ApoA1**
	Sample dilution	1000-fold	10-fold	1000-fold	10-fold	1000-fold
	AUC	0,75	0,7	0,52	0,78	0,69
	Cut-off	>0,015	>0,005	>0,165	>0,035	<0,23
	Sensitivity(%)	58	75	36	44	92
	Specificity(%)	90	60	80	100	50
	Median Ratio of patients	0.02	0.04	0.085	0.03	0.15
	Not	<0.02	<0.04	<0.085	<0.03	>0.15
	Intermediate	0.02-0.04	0.04-0.08	0.085-0.17	0.03-0.06	0.15-0.075
	Strong	>0.04	>0.08	>0.17	>0.06	<0.075
South-Korea (Serum)	**Group**	**N**	**BI (Mean)**	**Age range (years)**	**Male/Female (%)**	**MDT**
	MB	19	5.2+	21 to 82	63/37	0 (0%)
	PB	6	0	23 to 68	100/0	0 (0%)
	HC	25	NA	12 to 85	44/56	NA
	C	24	NA	24 to 95	71/29	NA
	ODD	24	NA	18 to 84	71/29	NA
	**Biomarker characteristics**	**αPGL-I IgM**	**IP-10**	**CRP**	**S100A12**	**ApoA1**
	Sample dilution	1000-fold	10-fold	1000-fold	10-fold	1000-fold
	AUC	0,77	0,6	0,67	0,6	0,54
	Cut-off	>0,03	>0,65	>0,205	>0,33	<0,105
	Sensitivity(%)	52	44	56	24	36
	Specificity(%)	100	92	79	96	100
	Median Ratio of patients	0.04	0.46	0.24	0.16	0.15
	Not	<0.04	<0.46	<0.24	<0.16	>0.15
	Intermediate	0.04-0.08	0.46-0.92	0.24-0.48	0.16-0.32	0.15-0.075
	Strong	>0.08	>0.92	>0.48	>0.32	<0.075
Bangladesh (FSB)	**Group**	**N**	**BI (Mean)**	**Age range (years)**	**Male/Female (%)**	**MDT**
	MB	11	1.4+	22 to 65	64/36	10 (91%)
	PB	16	0	17 to 70	38/62	2 (12,5%)
	HC	15	NA	17 to 60	53/47	NA
	**Biomarker characteristics**	**αPGL-I IgM**	**IP-10**	**CRP**	**S100A12**	**ApoA1**
	Sample dilution	1000-fold	10-fold	1000-fold	10-fold	1000-fold
	AUC	0,55	0,64	0,56	0,6	0,54
	Cut-off	>0.005	>0.175	> 0.375	> 1.06	< 0.225

The percentages of males and females, individuals receiving multidrug therapy (MDT), the mean bacteriological index (BI) and the age range of the multibacillary (MB) and paucibacillary (PB) patients, household contacts (HC), healthy (endemic) controls ((E)C) and patients with other dermatological diseases (ODD) in the study cohorts from Bangladesh (plasma and fingerstick blood (FSB)) and South Korea (serum). Ratio (R) values for αPGL-I IgM, IP-10, CRP, S100A12 and ApoA1 determined by the MBT strip in these samples were used to calculate the optimal cut-off (as determined by Youden's index) to discriminate leprosy patients from controls applying the indicated dilution.

The MBT readout (R-value) was also stratified based on the association with disease as strongly, intermediately or not associated. These categories are based on the patient median (Median).
Strong association: R ≥ 2x median of patient group, intermediate association: median of patient group ≤ R < 2x median of patient group, no association: R < median of patient group.

AUC: Area under the curve (as determined by computing receiver operating characteristic curves for leprosy patients compared to controls) . NA = not applicable

Supplementary Figures

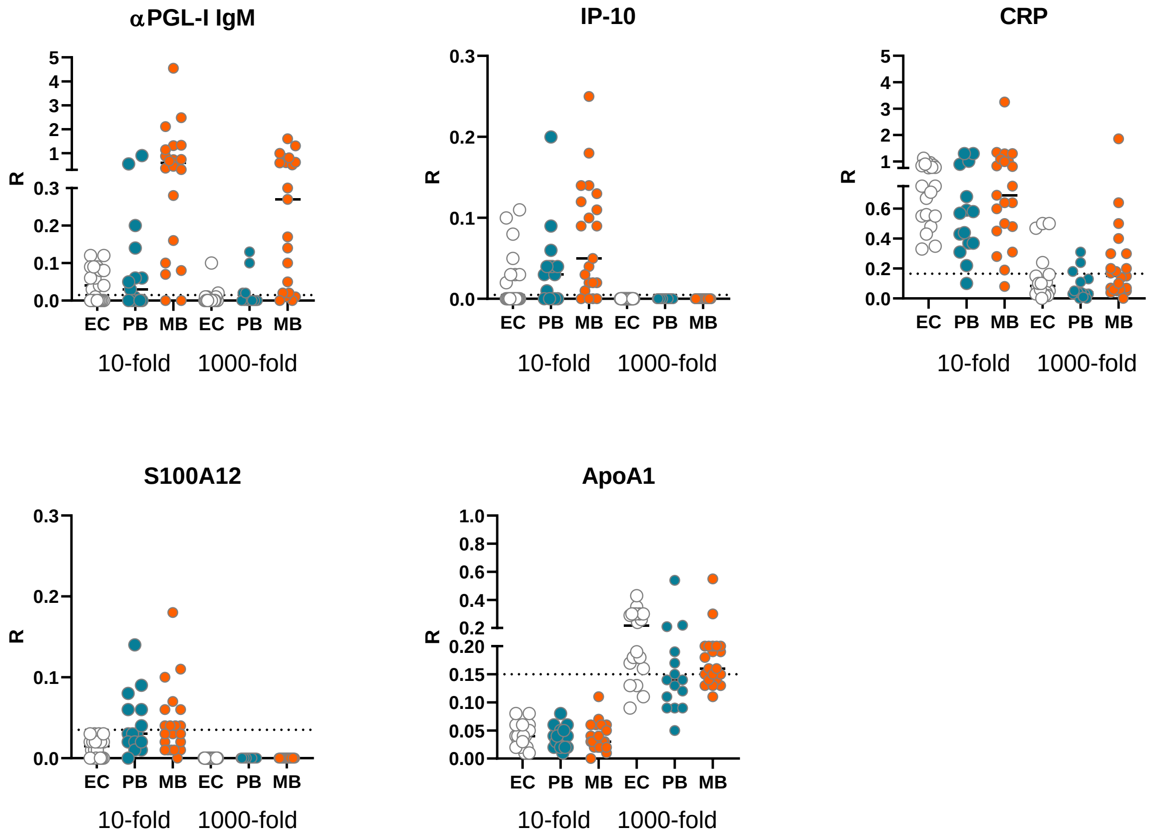

Supplementary Figure S1: Levels of αPGL-I IgM, IP-10, CRP, S100A12 and ApoA1 measured by multibiomarker test (MBT) strips (Bangladesh cohort) related to Figures 2 -4.

The levels of αPGL-I IgM, IP-10, CCL4, CRP, S100A12 and ApoA1 were assessed by applying 10- and 1000-fold diluted plasma samples of leprosy patients (n=36) and endemic controls (EC; n=20) to MBT strips. CCL4 was not detected in these plasma samples, a graph was therefore not included. Multibacillary patients (MB; n=21) are indicated with orange dots and paucibacillary patients (PB; n=15) with blue dots. Ratio values (R) for each biomarker were calculated by dividing the peak area of the test line by the peak area of the flow control line (*y*-axis). The dashed line indicates the optimal study cut-off value to discriminate leprosy patients from controls, determined by the Youden's index for the optimal dilution (10-fold: IP-10, S100A12; 1000-fold αPGL-I IgM, CRP, ApoA1.

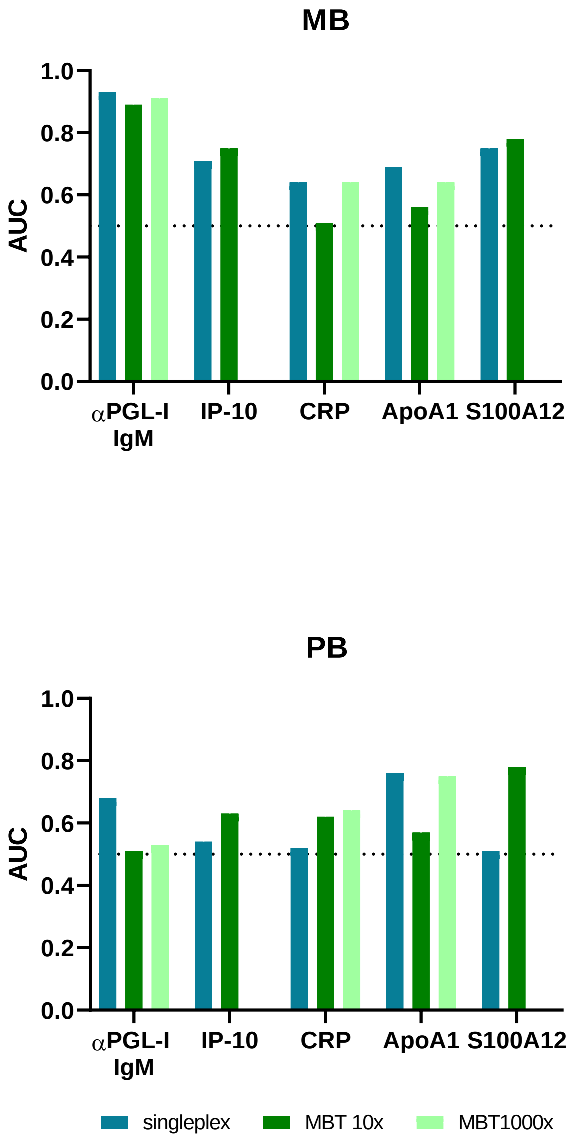

Supplementary Figure S2: Areas under the curve (AUCs) for αPGL-I IgM, IP-10, CRP, S100A12 and ApoA1 related to Figure 2.

Receiver operating characteristic (ROC) curves were computed and the respective AUC was calculated. AUCs obtained using either UCP-LFA strips specific for a single biomarker (singleplex) or multibiomarker test (MBT) strips were determined as a measure for discrimination between leprosy patients and endemic controls (EC). αPGL-I IgM, IP-10, CRP, S100A12 and ApoA1 were assessed in plasma samples of leprosy patients (MB=21; PB=15) and EC (n=20). The AUCs (*y*-axis) were calculated based on the Ratio values for each marker for singleplex strips (blue) or MBT strips in the 10-fold (dark green) and 1000-fold (mint green) dilution. Singleplex data was described previously, samples were diluted 10-fold (IP-10), 100-fold (αPGL-I IgM and S100A12), 1000-fold (CRP) and 10000-fold (ApoA1). The dashed line at 0.5 indicates a non-discriminatory AUC.

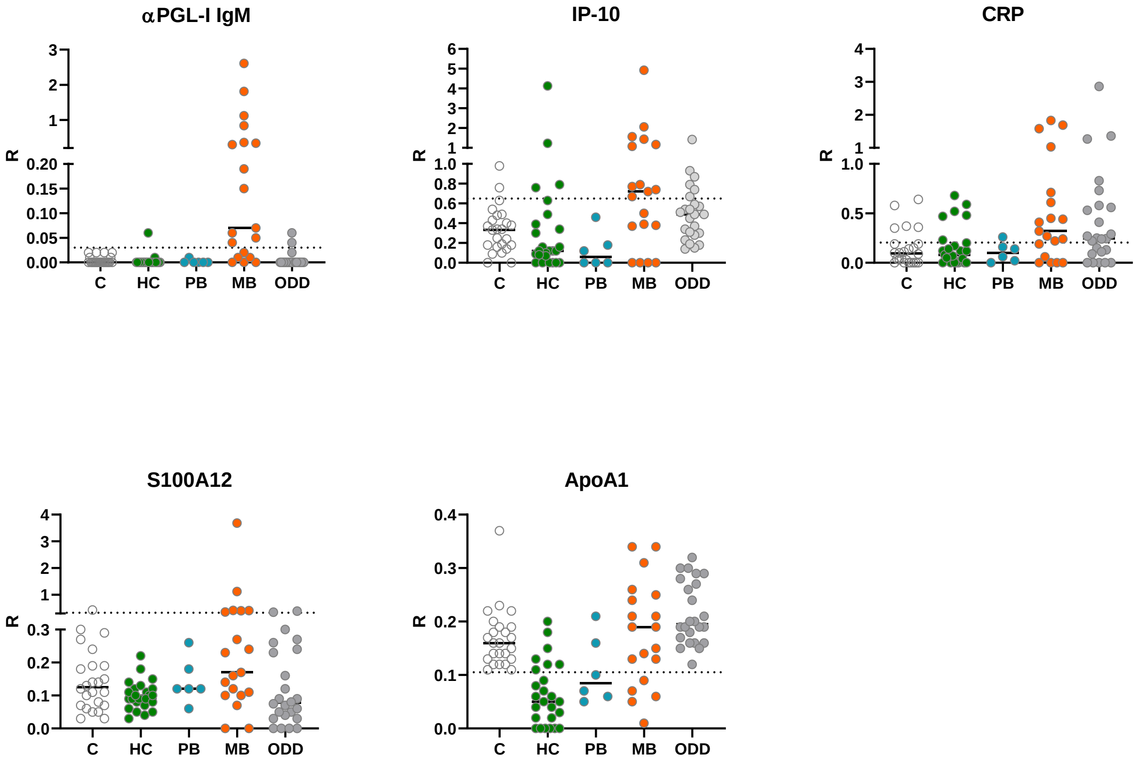

Supplementary Figure S3: Levels of αPGL-I IgM, IP-10, CRP, S100A12 and ApoA1 measured by multibiomarker test (MBT) strips (South Korea cohort) related to Figures 2-4.

The levels of αPGL-I IgM, IP-10, CRP, S100A12 and ApoA1 were assessed in serum samples of leprosy patients (n=25), household contacts (HC; n=25), healthy controls (C; n=24) and patients with other dermatological diseases (ODD; n=24) from South Korea using MBT strips. Results of the optimal dilution per biomarker are shown, 10-fold for IP-10 and S100A12 and 1000-fold for CRP, ApoA1 and αPGL-I IgM. Multibacillary patients (MB; n=19) are indicated with orange dots and paucibacillary patients (PB; n=6) with blue dots. Ratio values (R) (*y*-axis) were calculated by dividing the peak area of the test line by the peak area of the flow control line. The dashed line indicates the optimal study cut-off value to discriminate leprosy patients from controls, determined by the Youden’s index.

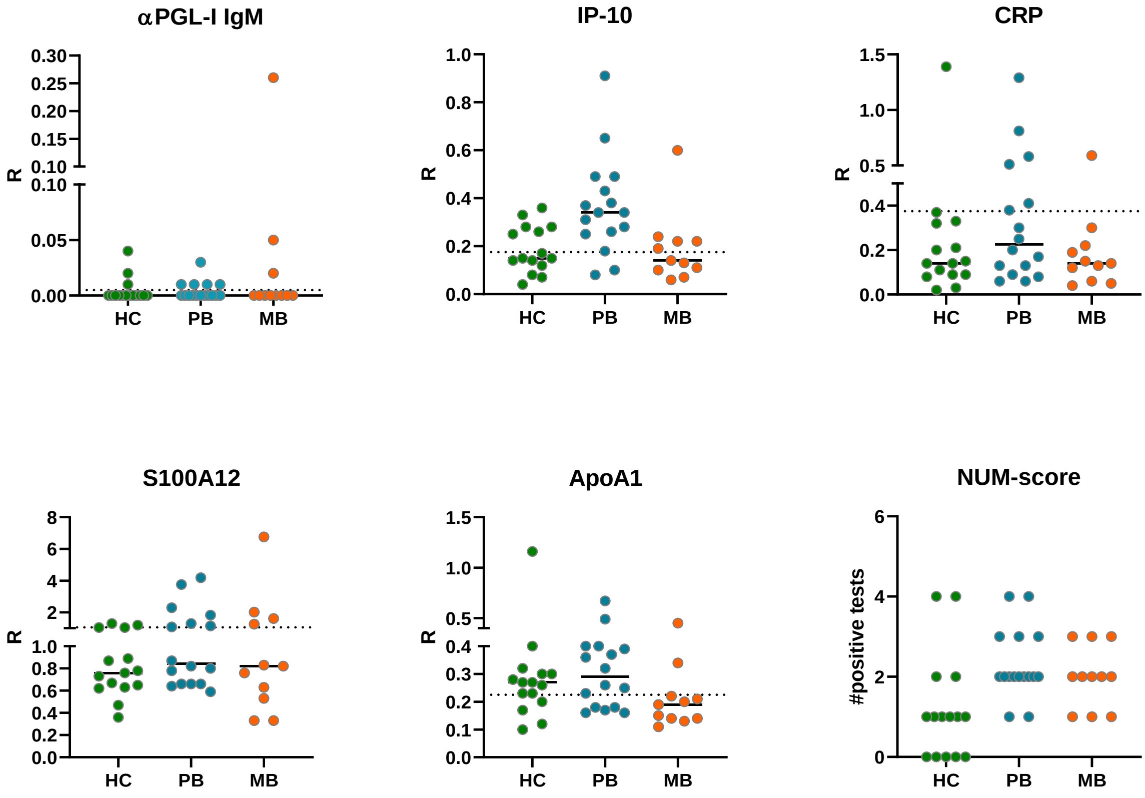

Supplementary Figure S4: Fingerstick blood (FSB) levels of αPGL-I IgM, IP-10, CRP, S100A12 and ApoA1 measured by MBT strips related to Figure 2.

The levels of αPGL-I IgM, IP-10, CRP, S100A12 and ApoA1 were assessed in FSB samples (two dilutions) of leprosy patients from Bangladesh (Leprosy; n=27) and household contacts (HC; n=15) using MBT strips. Patients treated for several months when FSB samples were taken (91% MB (n=10), 12,5% PB (n=2)) were included. Results of the optimal dilution per biomarker are shown, 10-fold for IP-10 and S100A12 and 1000-fold for CRP, ApoA1 and αPGL-I IgM. Ratio values (R) (*y*-axis) were calculated by dividing the peak area of the test line by the peak area of the flow control line. MBT NUM-scores were determined for HC, PB and MB patients. The dashed line indicates the optimal study cut-off value to discriminate leprosy patients from controls, determined by the Youden's index.

Transparent Methods

Study participants: Leprosy was diagnosed based on clinical, histological and bacteriological observations and classified as MB or PB as described by the WHO (World Health Organisation, 2019) and the bacteriological index (BI) was determined (Richardus et al., 2013). In Bangladesh 63 leprosy patients, 15 household contacts (HC) and 20 endemic controls (EC) were recruited between January 2013 and 2019 in leprosy endemic areas in Bangladesh as part of the MALTALEP/IDEAL trial (Richardus et al., 2013, van Hooij et al., 2020). Staff of leprosy- or TB clinics were excluded as EC (Supplementary Table S1). In South Korea participants included 25 leprosy patients, 25 frequent contacts of the patients (HC: 88% family contact, 12% office contact), 24 individuals with a history of other dermatological diseases (ODD) and 24 healthy controls from the same area (C) (Supplementary Table S1). ODD showed symptoms similar to leprosy, including patients with psoriasis vulgaris, eczema, fungal infections and sarcoidosis.

Ethics: This study was performed according to the Helsinki Declaration (2008 revision) and the study protocol was approved by the National Research Ethics Committee (Bangladesh Medical Research Council) (Ref no. BMRC/NREC/2010-2013/1534). Participants were informed about the study objectives, the samples and their right to refuse to take part or withdraw from the study without consequences for their treatment. Written informed consent was obtained before enrolment. All patients received treatment according to national guidelines.

Leprosy prevalence: The prevalence in the four Bangladeshi districts (Nilphamari, Rangpur, Panchagar and Thakurgaon) was 0.9 per 10,000 and the new case detection rate 1.18 per 10,000 (Rural health program, the leprosy mission Bangladesh, yearly district activity report 2018). The leprosy prevalence in South Korea was 0.025 per 10,000 (2018 (World Health Organisation, 2019)).

Samples: Plasma and whole blood assay samples were collected in Bangladesh, shipped to the LUMC on dry ice and stored at -80 ^{0}C until further testing (Khadge et al., 2015). For the WBA, 4ml venous blood was drawn and 1ml was applied directly to a microtube precoated with 10 µg *M. leprae* whole cell sonicate (WCS) or without stimulus (Med). After 24 h incubation at 37◦C the microtube was frozen at −20◦C. Serum samples from South Korea were collected and stored at -80 ^{0}C until testing by local health care workers. An extensive standard operating procedure and a quality control sample were provided to limit procedural differences. FSB was collected using disposable 50 µl Minivette® collection tubes (Heparin coated; Sarstedt) and directly mixed with 455 µl high salt finger stick (HSFS) buffer supplemented with 1% (v/v) Triton X-100 (HSFS;100mM Tris pH 8, 270mM NaCl, 1% (w/v) BSA). FSB was tested directly after collection in a reference center for leprosy patients in Bangladesh (The Leprosy Mission International, Bangladesh, Nilphamari Hospital).

Multi-biomarker test (MBT) production: MBT strips were assembled by mounting 10 mm glass fiber sample/conjugate pad (Glass Fiber Conjugate Pad #8964, Ahlstrom), 25 mm laminated nitrocellulose membrane (Sartorius UniSart CN95) and 10 mm absorbent pad (High Purity Cotton Grade #320, Ahlstrom) on a 36 mm plastic backing card; all strip materials were obtained from Kenosha (Amstelveen, the Netherlands). Sample/conjugate and absorbent pad overlap respectively 3 and 6 mm with the nitrocellulose. Nitrocellulose was pre-striped such that each MBT strip contained 6 Test (T) lines with respective Flow Control (FC) lines, providing capture zones for the six biomarkers. The six pairs of capture lines (T and FC) were distributed evenly over the 50 mm wide MBT strip starting at 14 mm from the base of the MBT strip. Capture lines were located in a linear array of slanted lines, such that liquid only passed single capture lines, with T and FC lines separated by 3 mm and a 5mm distance between individual T and FC pairs. Sample flow direction and scanning direction of the reader are perpendicular. Each Test (T_n) line comprised 300 ng capture molecules (Figure 1): T_1 = ND-

O-HSA, obtained through the Biodefense and Emerging Infections Research Resources Repository (https://www.beiresources.org/) (Cho et al., 1983); T_2 = mouse-anti-IP-10 mAb (BC-55, Diaclone Research, Besancon, France); T_3 = mouse-anti-CRP mAb (C5, Labned.com, Amstelveen, Netherlands); T_4 = mouse-anti-CCL4 mAb (MAB271, R&D systems, Minneapolis, USA);T_5 = goat-anti-S100A12 pAb (AF1052; R&D systems); and T_6 = Goat-anti-ApoA1 pAb (AF3664; R&D systems). The respective Flow Control (FC_n) lines comprised 37.5 ng Goat-anti-Mouse ($FC_{2,3}$), 75 ng Goat-anti-Rabbit (FC_6) or Rabbit-anti-Goat ($FC_{1,4,5}$) antibody per 6 mm (Sigma-Aldrich, Saint-Louis, United States). Individual UCP conjugates (Corstjens et al., 2014) were prepared with mouse-anti-IP-10 (BC-50; Diaclone Research), mouse-anti-CRP (CRP135; Labned.com), goat-anti-CCL4 (AF-271-NA; R&D systems), goat-anti-human IgM (I0759; Sigma-aldrich), goat-anti-S100A12 pAb (AF1052; R&D systems, Minneapolis, USA) and Rabbit-anti-ApoA1 (Clone # 2083A; R&D systems) at a concentration of 50 μg antibody per mg UCP. Sodium yttrium fluoride upconverting nanomaterials (200 nm, $NaYF_4:Yb^{3+},Er^{3+}$) functionalized with polyacrylic acid were obtained from Intelligent Material Solutions Inc. UCP reporter conjugates were applied to the sample/conjugate pad at a density of 400 ng per 7 mm. After assembly a quality control was performed, testing the MBT strips with recombinant proteins and standardized sample.

MBT assay: Banked plasma and serum samples were thawed and 50 μl was mixed with 455 μl HSFS buffer to obtain a 10-fold dilution similar to the dilution of the FSB sample upon collection. A 1000-fold dilution was prepared by adding 5 μl of the 10-fold dilution with 495 μl HSFS buffer (1000-fold dilution). The diluted samples (500 μl) were added to a channel in a disposable tray, immunochromatography was initiated by placing the MBT strip into the channel and allowed to continue for at least 30 min. In South Korea this procedure was performed by local health care workers without prior training. Air dried MBT strips were scanned with a portable reader (ESEQuant *LFR* reader with 980 nm excitation and 550 nm

emission; QIAGEN Lake Constance GmbH, Stockach, Germany). The strip holder was in-house adapted to fit the MBT which requires scanning perpendicular to the sample flow. Test results are displayed as the Ratio (R) value of the signal (peak area) from individual T_n lines normalized to the respective FC_n signal measured at the respective lines as determined by LF-Studio (ver. 3.3.8; QIAGEN Lake Constance GmbH) (Figure 1).

Data analysis: The R-value corresponds to the level of the biomarker present in the sample. Two different scores based on the MBT readout were evaluated.

i) **NUM-score**: Based on R-values, the optimal cut-offs to discriminate leprosy patients from their respective controls were determined using the Youden's index (Fluss et al., 2005) per biomarker (Supplementary Table S1). Qualitative stratification of the biomarker result as positive or negative based on these cut-offs was used to calculate the number of positive biomarkers in the MBT per individual.

ii) **ALGO-score**: The ALGO-score was calculated using the median R-values of the individual biomarkers for the leprosy patients. First, scores were classified for each biomarker in three groups based on the association with disease as strong, intermediate or not associated (Supplementary Table S1). Strong association: $R \geq 2x$ median of patient group, intermediate association: median of patient group $\leq R < 2x$ median of patient group, no association: $R <$ median of patient group. Second, the ALGO-score was set as 2x the number of strong biomarkers (2x n_{strong}), plus the number of intermediate biomarkers ($n_{intermediate}$), minus the number of biomarkers not associated with disease (n_{not}) .

Note that for ApoA1, the 1000-fold dilution resulted in an inverse correlation of the Ratio values with the biomarker level a consequence of a distinct but reproducible high dose hook effect.

Statistical analysis was performed using GraphPad Prism version 8.0.1 (GraphPad Software, San Diego,CA, USA; http://www.graphpad.com). Receiver operating characteristic (ROC)

curves were computed in Graphpad Prism and the respective area under the curve (AUC) was calculated. Group differences were determined using Mann-Whitney U test. The statistical significance level used was $p \leq 0.05$.

References

Cho, S. N., Yanagihara, D. L., Hunter, S. W., Gelber, R. H. and Brennan, P. J. (1983) 'Serological specificity of phenolic glycolipid I from Mycobacterium leprae and use in serodiagnosis of leprosy', *Infect Immun,* 41(3), pp. 1077-83. doi:

Corstjens, P. L., de Dood, C. J., Priest, J. W., Tanke, H. J. and Handali, S. (2014) 'Feasibility of a lateral flow test for neurocysticercosis using novel up-converting nanomaterials and a lightweight strip analyzer', *PLoS Negl Trop Dis,* 8(7), pp. e2944. doi: 10.1371/journal.pntd.0002944

Fluss, R., Faraggi, D. and Reiser, B. (2005) 'Estimation of the Youden Index and its associated cutoff point', *Biom J,* 47(4), pp. 458-72. doi:

Khadge, S., Banu, S., Bobosha, K., van der Ploeg-van Schip, J. J., Goulart, I. M., Thapa, P., Kunwar, C. B., van Meijgaarden, K. E., van den Eeden, S. J., Wilson, L., Kabir, S., Dey, H., Goulart, L. R., Lobato, J., Carvalho, W., Bekele, Y., Franken, K. L., Aseffa, A., Spencer, J. S., Oskam, L., Otttenhoff, T. H., Hagge, D. A. and Geluk, A. (2015) 'Longitudinal immune profiles in type 1 leprosy reactions in Bangladesh, Brazil, Ethiopia and Nepal', *BMC Infect Dis,* 15, pp. 477. doi: 10.1186/s12879-015-1128-0

Richardus, R. A., Alam, K., Pahan, D., Feenstra, S. G., Geluk, A. and Richardus, J. H. (2013) 'The combined effect of chemoprophylaxis with single dose rifampicin and immunoprophylaxis with BCG to prevent leprosy in contacts of newly diagnosed leprosy cases: a cluster randomized controlled trial (MALTALEP study)', *BMC Infect Dis,* 13, pp. 456. doi: 10.1186/1471-2334-13-456

van Hooij, A., Tió-Coma, M., Verhard, E. M., Khatun, M., Alam, K., Tjon Kon Fat, E., de Jong, D., Sufian Chowdhury, A., Corstjens, P., Richardus, J. H. and Geluk, A. (2020) 'Household Contacts of Leprosy Patients in Endemic Areas Display a Specific Innate Immunity Profile', *Front Immunol,* 11, pp. 1811. doi: 10.3389/fimmu.2020.01811

World Health Organisation (2019) 'Global leprosy update, 2018: moving towards a leprosy-free world', *Wkly Epidemiol Rec,* 94(35/36), pp. 389-412. doi:
